# A dataset of chest X-ray reports annotated with Spatial Role Labeling annotations

**DOI:** 10.1016/j.dib.2020.106056

**Published:** 2020-07-25

**Authors:** Surabhi Datta, Kirk Roberts

**Affiliations:** School of Biomedical Informatics, The University of Texas Health Science Center, Houston, TX, USA

**Keywords:** Spatial relations, Spatial Role Labeling, Radiology report, Chest radiology, Natural language processing, Information extraction

## Abstract

In this paper, we present a dataset consisting of 2000 chest X-ray reports (available as part of the Open-i image search platform) annotated with spatial information. The annotation is based on Spatial Role Labeling. The information includes annotating a radiographic finding, its associated anatomical location, any potential diagnosis described in connection to the spatial relation (between finding and location), and any hedging phrase used to describe the certainty level of a finding/diagnosis. All these annotations are identified with reference to a spatial expression (or Spatial Indicator) that triggers a spatial relation in a sentence. The spatial roles used to encode the spatial information are Trajector, Landmark, Diagnosis, and Hedge. In total, there are 1962 Spatial Indicators (mainly prepositions). There are 2293 Trajectors, 2167 Landmarks, 455 Diagnosis, and 388 Hedges in the dataset. This annotated dataset can be used for developing automatic approaches targeted toward spatial information extraction from radiology reports which then can be applied to numerous clinical applications. We utilize this dataset to develop deep learning-based methods for automatically extracting the Spatial Indicators as well as the associated spatial roles [1].

Specifications Table

**Table:** 

Subject	*Health Informatics*
Specific subject area	*Spatial information extraction from chest X-ray reports based on Spatial Role Labeling schema for spatial language understanding in radiology reports*
Type of data	*Table, Figure, Text, Annotated data in XML format*
How data were acquired	*A subset of 2000 chest X-ray reports were used from a pool of 3996 de-identified reports collected from the Indiana Network for Patient Care (available as one of the Open-i datasets released by the National Library of Medicine.)*
Data format	*Raw, Processed*
Parameters for data collection	*2000 chest X-ray reports that are annotated with important spatial information were selected from the set of 2470 non-normal reports in the Open-i chest X-ray report dataset as adjudicated by two annotators.*
Description of data collection	*These 2000 reports were annotated with four spatial roles using the Brat toolkit. First, the spatial indicators (usually the spatial prepositions) triggering any spatial relation between a radiographic finding and an anatomical location were annotated for each sentence. Then, four spatial roles–the radiographic finding, its corresponding location, hedging phrase, and any potential diganosis were annotated with respect to a specific spatial indicator.*
Data source location	*Primary data source: Open-i chest X-ray dataset* (https://openi.nlm.nih.gov/). *Associated research paper: “Preparing a collection of radiology examinations for distribution and retrieval”*–https://doi.org/10.1093/jamia/ocv080
Data accessibility	Repository name: Mendeley data repository Data identification number: 10.17632/yhb26hfz8n.1 Direct URL to data: https://doi.org/10.17632/yhb26hfz8n.1, https://github.com/krobertslab/datasets/tree/master/rad-sprl
Related research article	*S. Datta, Y. Si, L. Rodriguez, S. E. Shooshan, D. Demner-Fushman, K. Roberts, Understanding spatial language in radiology: Representation framework, annotation, and spatial relation extraction from chest X-ray reports using deep learning, Journal of Biomedical Informatics 108 (2020) 103473. doi:10.1016/j.jbi.2020.103473.*

**Value of the Data**

• The spatial information annotated in this dataset captures clinically significant information of chest X-ray imaging results. This annotation schema proposes a way to encode radiological spatial knowledge from report text. The annotated information includes the main radiographic finding detected, the anatomical location where the finding has been described to be present, any diagnosis associated with the finding-location pair, as well as any hedging phrase used to suggest the diagnosis or the finding.
• The dataset can be used to develop automatic NLP systems for extracting spatial information from radiology reports. These systems have the potential to facilitate various clinical applications. A few of these include easy visualization of contextual information associated with abnormal radiographic findings from a spatial perspective, automatic tracking of findings, and automatic annotation of corresponding radiographic images with spatial and diagnosis information.
• The models developed on this dataset could be further leveraged by applying them on other types of radiology reports belonging to different imaging modality such as chest Computed Tomography (CT) scans and Magnetic Resonance Imaging (MRI) as the annotated information types are common across different modalities and/or anatomies.

## 1 Data description

**Fig. 1:**
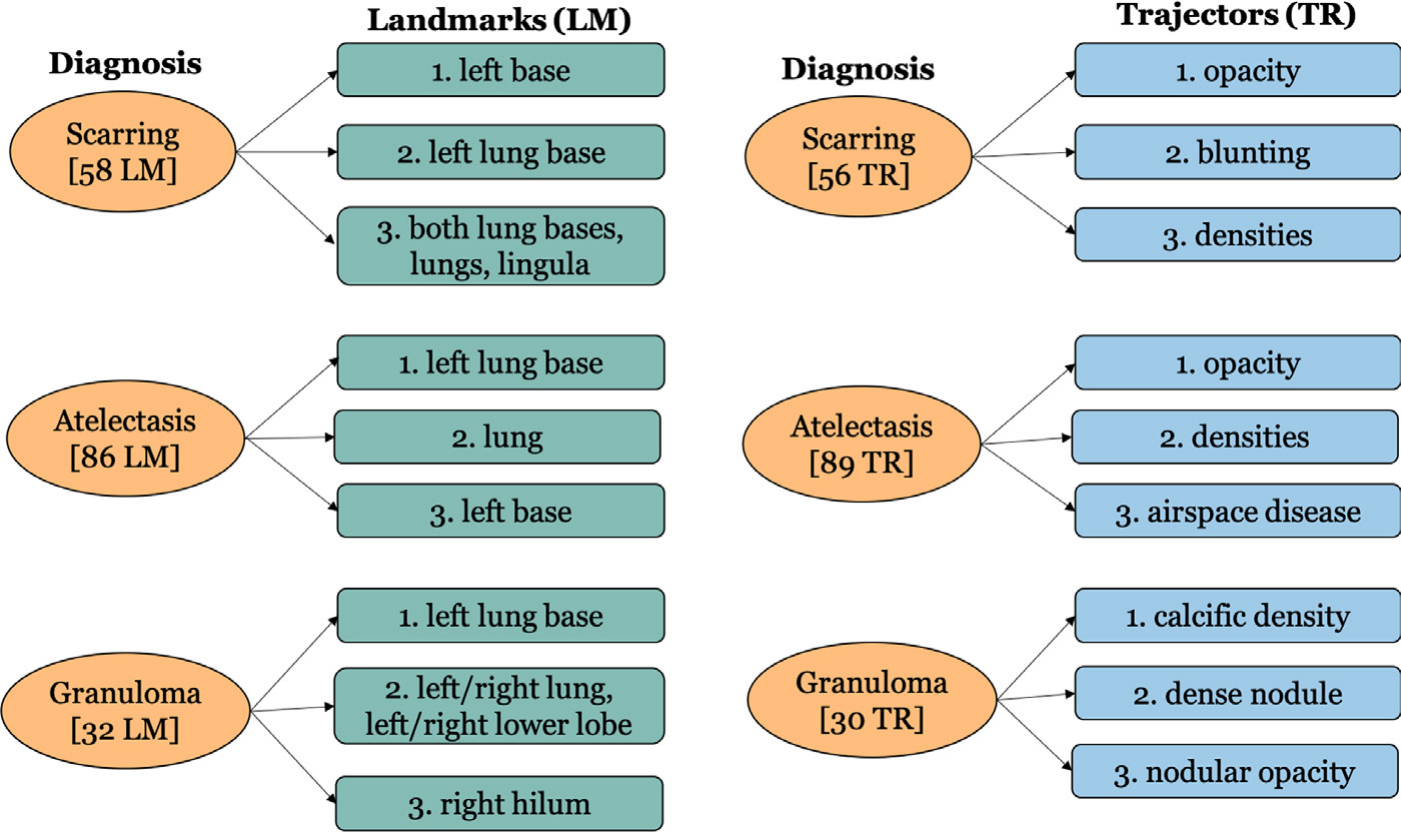
Most common associated landmarks and trajectors for three frequent Diagnosis. [*n* LM] indicates that a particular diagnosis is connected to a total of *n* landmarks, while [*n* TR] indicates that a particular diagnosis is connected to a total of *n* trajectors.

**Fig. 2:**
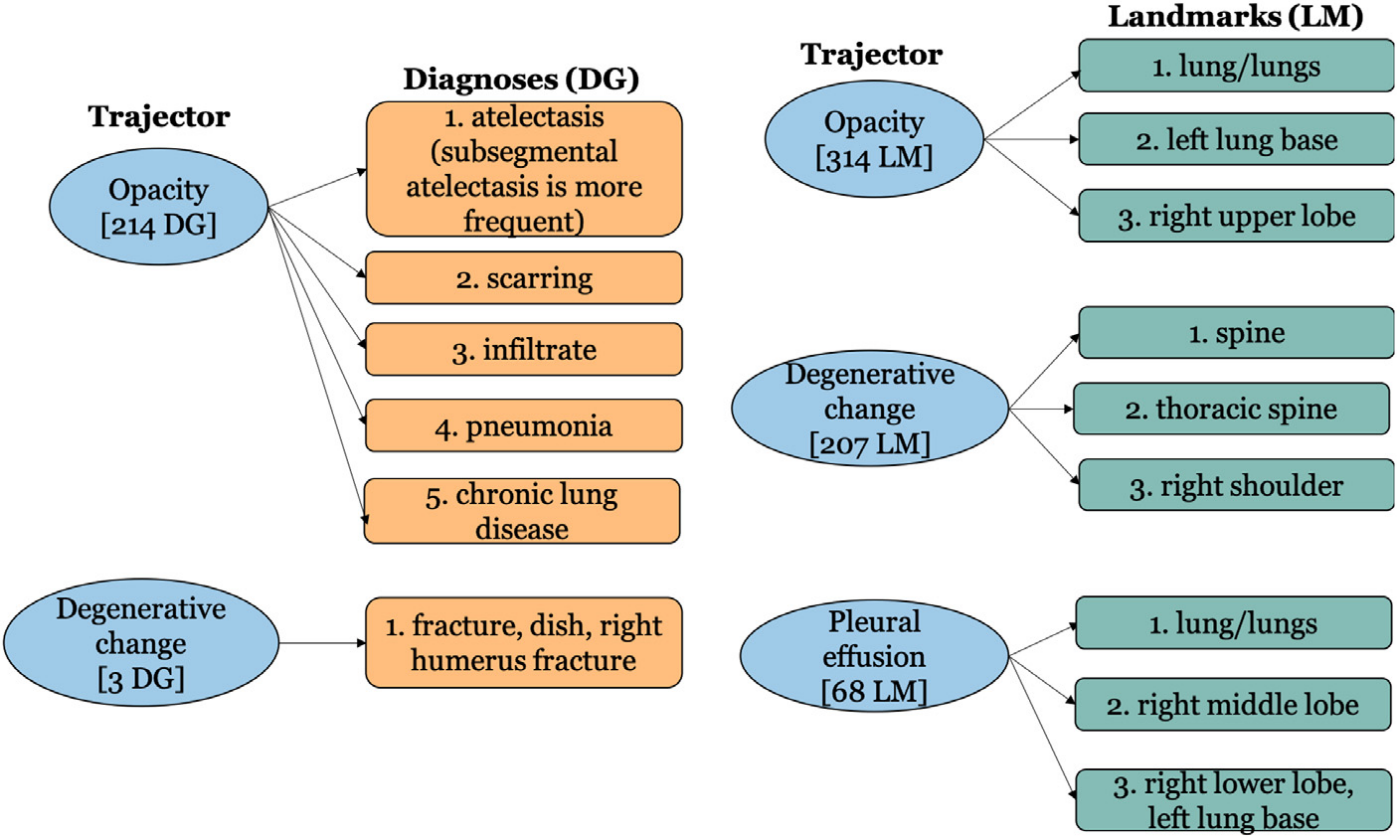
Most common associated diagnoses and landmarks for three frequent Trajector. [*n* DG] indicates that a particular trajector is connected to a total of *n* dagnoses, while [*n* LM] indicates that a particular trajector is connected to a total of *n* landmarks.

**Fig. 3:**
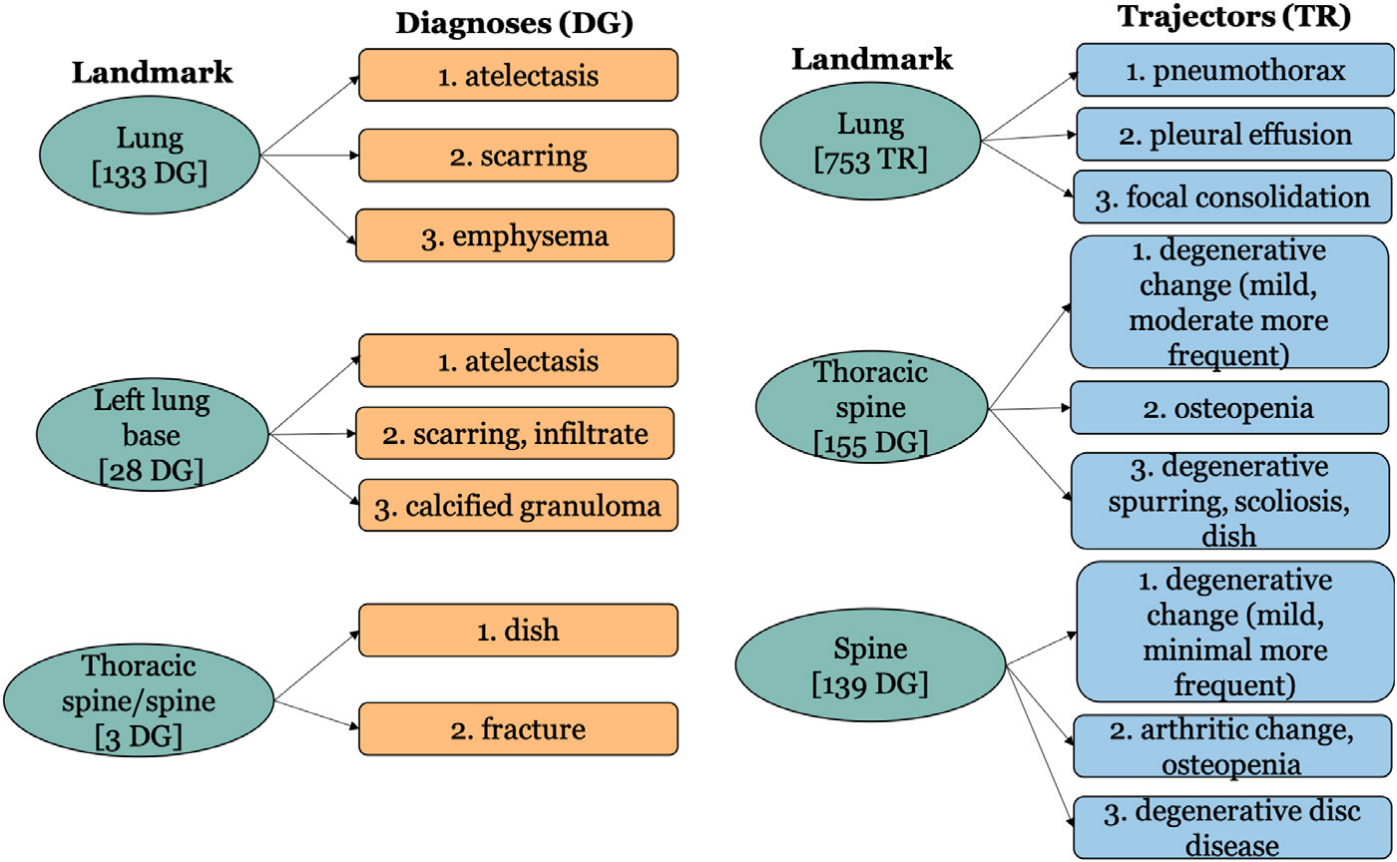
Most common associated diagnoses and trajectors for three frequent Landmark. [*n* DG] indicates that a particular landmark is connected to a total of *n* dagnoses, while [*n* TR] indicates that a particular landmark is connected to a total of *n* trajectors.

This 2000 chest X-ray reports dataset is a subset of 3996 reports collected from the Indiana Network for Patient Care [2]. Specifically, the 2000 report subset is composed from the set of 2470 non-normal reports as judged by two human annotators. The annotation schema is based on Spatial Role Labeling (SpRL) [3], [4] and has been extended to encode information in radiology context. This includes identifying a Spatial Indicator in a sentence and consequently annotating the main radiographic finding and anatomical location that are connected by this Spatial Indicator. Additionally, the spatial annotations include any potential diagnosis identified in a sentence with reference to the spatial relation between a finding and a location. The annotations also include any uncertainty phrase or hedge used to describe a finding/diagnosis. These four information types denote the four spatial roles with respect to a Spatial Indicator in a sentence. The schema is referred to as Rad-SpRL. The dataset is included in XML format (available at https://doi.org/10.17632/yhb26hfz8n.1 in the Mendeley data repository and https://github.com/krobertslab/datasets/) and the relevant details are described in Table 1. A few details of the Spatial Indicators in the dataset are included in Table 2. In total, there are 29 unique spatial expressions. The most frequent phrases for each of the four spatial roles annotated are shown in Table 3. We also note the frequent descriptors used in describing roles like Trajector and Diagnosis. Note that ‘*XXXX*’ is used to denote any de-itentified term in the report text. For each of Diagnosis, Trajector, and Landmark, the most common associated other two spatial roles are demonstrated in Figs. 1–3. We provide a brief statistics on the terms that are annotated as two different spatial roles depending on the context in a sentence in Table 4. We also analyze the terms expressing Hedge role (illustrated in Table 5).

**Table 1:** Annotated dataset descriptions.

**Attribute**	**Description**
Document	Represents a chest X-ray report
Text	Raw text of the report
Annotations	Contains the processed text and spatial annotations for a report
Token	Contains start character and number of characters of a token
Sentence	Contains start token number and number of included tokens to identify a sentence
RadSpRLRelation	Indicates the presence of a spatial relation. Includes the start token number and number of tokens of a spatial expression (Spatial Indicator) in a sentence, also contains all the associated spatial roles with respect to this Spatial Indicator

**Spatial roles under RadSpRLRelation**	

Trajector	Radiological entity (usually a radiographic finding whose position is described
Landmark	Anatomical location of a Trajector
Diagnosis	Potential diagnosis associated with a spatial relation
Hedge	Any uncertainty phrase used to describe a finding or diagnosis

**Table 2:** Spatial indicator statistics.

**Parameter**	**Frequency**
Total number of Spatial Indicators	1962
Number of distinct Spatial Indicators	29

**Most frequent indicators**	

*of*	765
*in*	526
*without*	176
*with*	141
*within*	102

**Table 3:** Most frequent terms for each spatial role.

**Spatial Role**	**Term**	**Frequency** (descriptors contained in Term)
Trajector	*opacity*	279 (nodular, streaky, interstitial, focal airspace, focal, airspace, vague, patchy, bibasilar, ill-defined, mild streaky, subtle increased, few small nodular, round, scattered, rounded nodular, abnormal, vague nodular, patchy airspace, bilateral, bandlike, vague patchy, dense, minimal, minimal streaky, streaky basilar, alveolar)
	*degenerative change*	205 (mild, minimal, diffuse, moderate, severe, multilevel, chronic, advanced)
	*pneumothorax*	63 (moderate right-sided, large)
	*pleural effusion*	63 (large, small bilateral, large right)
	*consolidation*	57 (focal, focal airspace, dense)

Total Distinct		861

Landmark	*lung*	285 (also includes lungs)
	*thoracic spine*	146 (mid, lower)
	*spine*	111
	*left lung base*	43
	*thorax*	40

Total Distinct		570

Diagnosis	*scarring*	53 (pleural, pleural-parenchymal, chronic)
	*atelectasis*	83 (subsegmental, focal, chronic subsegmental, foci of subsegmental, lingular)
	*infiltrate*	21 (focal)
	*granuloma*	15 (calcified, partially calcified)
	*emphysema*	11

Total Distinct		224

hedge	*may represent*	40
	*XXXX*	39
	*consistent with*	38 (focal)
	*XXXX represent*	34 (also includes XXXX represents, XXXX representing, XXXX representative of)
	*compatible with*	21
Total Distinct		80

**Table 4:** Overlapping terms between two spatial roles.

**Parameter**	**Frequency**
Distinct overlapping terms (Trajector and Diagnosis)	45
Distinct overlapping terms (Trajector and Landmark)	73
Same terms with equal frequency (Trajector and Landmark)	38

**Terms appearing more as****Trajector****and less as****Diagnosis**	

**Term**	**Frequency**

*infiltrate/focal infiltrate*	Trajector:40 Diagnosis:19
*calcified granuloma/calcified granulomas*	Trajector:37 diagnosis:6
*focal airspace disease*	Trajector:32 Diagnosis:2
*bronchovascular crowding*	Trajector:22 Diagnosis:2
*fracture/fractures*	Trajector:18 Diagnosis:3
*nodule*	Trajector:18 Diagnosis:2

**Terms appearing more as****Diagnosis****and less as****Trajector**	

**Term**	**Frequency**

*scarring*	Diagnosis:44 Trajector:21
*atelectasis*	Diagnosis:43 Trajector:14
*subsegmental atelectasis*	Diagnosis:23 Trajector:7
*emphysema*	Diagnosis:9 Trajector:4

**Terms appearing more as****Landmark****and less as****Trajector**	

**Term**	**Frequency**

*right upper lobe*	Landmark:35 Trajector:2
*right*	Landmark:27 Trajector:2
*right base*	Landmark:15 Trajector:2

**Common terms appearing both as****Trajector****and****Landmark****with equal frequency**	

**Term**	**Frequency**

*osseous structures*	31
*region*	5
*peripheral aspect*	4

**Table 5:** Analysis of Hedge terms.

**Description**	**Terms**
Frequent Hedges that appear without Diagnosis	*possible/possibly, or, probable/probably, appears to be, versus*
Frequent Hedges that appear with Diagnosis	*may represent, consistent with, XXXX, compatible with, XXXX representing*
Hedges that only appear when there is no Diagnosis	*apparent, questionable, and/or, probable, suggestion of, difficult to exclude, or XXXX, or, approximately, apparently, cannot be excluded* (11)
Example Hedges that only appear once	*may be partially due to, favored to represent, cannot be excluded, raise concern for, difficult to exclude*

## 2 Experimental design, materials and methods

In this dataset, we attempt to widen the scope of clinically significant information types to be extracted from chest X-ray reports and additionally aim to relate all the information in context to a spatial relation between a finding and a location. This provides more contextual information about a radiographic finding. Many of the previous works on radiology information extraction mainly focused on extracting radiological entities (findings, diagnoses, etc.) separately without establishing any relation among these entities [5], [6], [8], [7].

We further analyze the variations of Spatial Indicators in the dataset. Besides the five most frequent ones mentioned in Table 2, the other spatial prepositions include – ‘*at*’, ‘*over*’, ‘*on*’, ‘*throughout*’, ‘*under*’, ‘*along*’, ‘*near*’, ‘*to*’, ‘*through*’, ‘*between*’, ‘*adjacent*’, ‘*beneath*’, ‘*from*’, ‘*into*’, ‘*below*’, ‘*above*’, ‘*around*’, ‘*towards*’, ‘*about*’, ‘*behind*’. This dataset also includes four more verbal spatial expressions – ‘*overlie*’, ‘*overlies*’, ‘*overlying*’, and ‘*involving*’. However, these four expressions occur very infrequently and together account for 30 out of 1962 Spatial Indicators. Also, note that the indicator ‘*without*’ denotes a negated spatial relation and is oftentimes present as part of the common negated phrase used in radiology reports – ‘*without evidence of*’.

We inspect the dataset to analyze the most frequent terms annotated for each spatial role and observe that the top five frequent Trajectors are different from the five most frequent Diagnosis terms (as illustrated in Table 3). There are more distinct Trajectors and Landmarks than Diagnosis and Hedge terms.

We also analyze, for each spatial role, the most frequently associated other roles (Figs. 1–3). For this, we consider three terms among the five most frequent terms (shown in Table 3) for each role. It is interesting to observe that no diagnoses are associated with three frequent radiographic findings (Trajectors) – ‘*pneumothorax*’, ‘*pleural effusion*’, and ‘*consolidation*’ (as shown in Fig. 2).

In the process of annotating the reports, we noticed that some terms take different spatial roles depending on the context. We then inspect this overlap between two spatial roles in our annotated dataset. Specifically, the overlapping characteristics between Trajector and Diagnosis as well as between Trajector and Landmark are shown in Table 4. There are more distinct terms that have overlap between Trajector and Landmark than between Trajector and Diagnosis. Around 52% of the terms that act as both Trajector and Landmark an equal number of times oftentimes have the same text span and are related to anatomical structures or portions. Consider the following example:

*Visualized **osseous structures**of the thorax are without acute abnormality.*

Here, ‘*osseous structures*’ act as both Trajector and Landmark. It takes the role of a Trajector when considered in relation to the indicator ‘*of*’ and acts as a Landmark when considered in relation to ‘*without*’.

Additionally, we note that the terms that are annotated as both Trajector and Landmark appear more often as a Landmark than a Trajector (as shown in Table 4). There are certain findings like ‘*pleural thickening*/*thickening*’ which appear both as Trajector and Diagnosis with the same frequency.

Since the hedging terms are used both in context to describing a radiographic finding as well as a diagnosis, we intend to investigate their distribution in both the cases. We find that certain phrases such as ‘*probable*’ and ‘*or*’ are more representative of describing the findings rather than diagnoses. We also witness a variety of hedging expressions that occur rarely in the dataset. Besides the ones presented in Table 5, few other rare hedging phrases include – ‘*possibly related to*’, ‘*is a consideration*’, ‘*favored as*’, ‘*could be secondary to*’, and ‘*cannot be ruled out*’.

## Ethics statement

This work includes chest X-ray reports of patients collected from the Indiana Network for Patient Care in a previous study [2]. The reports are de-identified and do not involve experimentation with human subjects.

## Declaration of Competing Interest

The authors declare that they have no known competing financial interests or personal relationships which have, or could be perceived to have, influenced the work reported in this article.
